# LAG3 and PD1 co-inhibitory molecules collaborate to limit CD8^+^ T cell signaling and dampen antitumor immunity in a murine ovarian cancer model

**DOI:** 10.18632/oncotarget.4751

**Published:** 2015-07-23

**Authors:** Ruea-Yea Huang, Cheryl Eppolito, Shashikant Lele, Protul Shrikant, Junko Matsuzaki, Kunle Odunsi

**Affiliations:** ^1^ Department of Gynecologic Oncology, Roswell Park Cancer Institute, Buffalo, New York, USA; ^2^ Department of Immunology, Roswell Park Cancer Institute, Buffalo, New York, USA; ^3^ Center for Immunotherapy, Roswell Park Cancer Institute, Buffalo, New York, USA; ^4^ Department of Research, Mayo Clinic, Scottsdale, Arizona, USA

**Keywords:** LAG3, PD1, antibody blockade, T cell signaling, ovarian cancer

## Abstract

The immune co-inhibitory receptors lymphocyte activation gene-3 (LAG3) and programmed cell death 1 (PD1) synergistically contribute to autoimmunity and tumor evasion. Here we demonstrate how they collaborate and interact to regulate T cell function. We first show that LAG3 and PD1 are co-expressed on both OVA-specific and non-specific T cells infiltrating murine ovarian tumors. Dual antibody blockade or genetic knockout of LAG3 and PD1 significantly enhanced T effector function and delayed tumor growth. LAG3 and PD1 co-localized in activated CD8^+^ T cells *in vitro* at the trans-Golgi vesicles, early/recycling endosomal compartments, lysosomes, and microtubule organizing center. Importantly, LAG3 and PD1 cluster with pLck at the immunological synapse. Reciprocal immunoprecipitation of T cell extracts revealed physical interaction between LAG3 and PD1. Mutational analyses indicate that the cytoplasmic domain of LAG3 is not absolutely required for its association with PD1, while the ITIM and ITSM of PD1 are necessary for its association with LAG3. Finally, LAG3 protein also associates with the Src-homology-2 domain-containing phosphatases (SHP1/2) which are known to be recruited by PD1 during T cell signaling. Our data indicate that the association of LAG3 with PD1 contributes to their rapid trafficking to the immunological synapse, leading to a synergistic inhibitory effect on T cell signaling.

## INTRODUCTION

Emerging evidence suggests that elevated expression of inhibitory receptors on tumor antigen-specific T cells is one of the mechanisms by which tumors evade host immune surveillance [1]. Although blockade of certain individual inhibitory receptor with specific antibodies has shown significant promise in overcoming immune suppression and mediating tumor regression [2–4], recent studies indicate that multiple inhibitory receptors (including CD160, KLRG-1, TIM-3, 2B4, BTLA and LAG3) are often co-expressed on tumor-antigen specific CD8^+^ T cells [5]. Of these, the cytotoxic T lymphocyte antigen 4 (CTLA-4) and programmed cell death 1 (PD1) have been studied most extensively in preclinical models and in clinical trials [2–4, 6, 7]. PD1 belongs to the CD28/CTLA-4 family and is expressed on the surface of activated T cells, B cells, and macrophages [8]. Interestingly, it has been demonstrated that dual simultaneous blockade of inhibitory receptors synergistically reverse the exhausted state of T cells and render them more functional than single blockade of the receptors [9–12]. Most recently, PD1 and CTLA-4 have been tested in combination in the clinic for melanoma and are more effective than either agent alone [13]. Because the synergistic blockade of these receptors are usually not simply additive, this raises the possibility that the co-inhibitory receptors may interact at the physical, biochemical and molecular levels, and thereby coordinately regulate the functional fate of T cells.

We have previously demonstrated that a subset of tumor antigen-specific CD8^+^ T cells co-expressing LAG3 and PD1 are impaired in IFN-γ and TNF-α production and that simultaneous blockade of LAG3 and PD1 restores effector function of human ovarian tumor antigen-specific T cells to a level that is above the additive effects of single blockade of LAG3 or PD1 alone [10]. In the current study, we focused on investigating how LAG3 and PD1 collaborate to negatively regulate CD8^+^ T cell immunity. In previous reports, although *Lag3^−/−^* mice develop increased CD4^+^ and CD8^+^ T cell islet infiltration and intra-islet proliferation, they exhibit only a minor autoimmune phenotype [14]. In contrast, PD1 knockout (*Pdcd1 ^−/−^*) mice, develop various types of autoimmune diseases depending upon the background [15]. Mice deficient in both LAG3 and PD1 proteins (*Lag3^−/−^*Pdcd1*^−/−^*) exhibit a wide range of severe autoimmune diseases [16] that leads to 80% of lethality at 4–12 weeks after birth and enhanced antitumor activity in murine tumor models [12]. Thus, while LAG3 and PD1 act synergistically to control immune homeostasis and mediate tumor-induced tolerance, the mechanism(s) of this synergy are currently unknown.

The PD1 cytoplasmic domain is known to contain immunoreceptor tyrosine-based inhibition motif (ITIM) and immunoreceptor tyrosine-based switch motif (ITSM) that engage signal-aborting tyrosine phosphatases such as SHP1 or SHP2 [17, 18]. Upon binding by PD1-ligand, PD1 limits T cell activation through the recruitment of SHP1 or SHP2 to attenuate T cell receptor signaling and inhibits cytokine production [19]. In contrast, little is known regarding the mechanism(s) by which LAG3 ligation blocks primary T cell activation. It has been demonstrated that LAG3 binds to MHC class II molecules [20] and possibly associates with the TCR:CD3 complex to negatively regulate TCR-induced signal transduction [21]. Unlike PD1, LAG3 does not contain a tyrosine-phosphorylation motif, nor the ITIM or ITSM. The LAG3 cytoplasmic domain has three regions that are conserved in humans and mice [22, 23]. The first region has a potential serine phosphorylation site, which may serve as a protein kinase C binding site [24]. The second is a conserved KIEELE motif that is required for LAG3-mediated immune inhibition [23, 25]. The third is an unusual glutamic acid-proline (EP) repetitive sequence that is found in molecules known to mediate signaling [22]. It has recently been demonstrated that the EP motif is important for the rapid translocation of LAG3 to the cell surface after stimulation [26]. In order to understand the molecular basis of how LAG3 and PD1 synergistically inhibit T cell function, we asked whether LAG3 interacts with PD1 or other molecules involved in proximal TCR signaling events. Our results indicate that while LAG3 and PD1 may function independently of each other, they closely associate and collaborate to mediate inhibition of TCR-signaling events.

## RESULTS

### CD8^+^ T cells from Lag3^−/−^Pdcd1^−/−^ knockout mice exhibit enhanced effector phenotype

**Figure 1:**
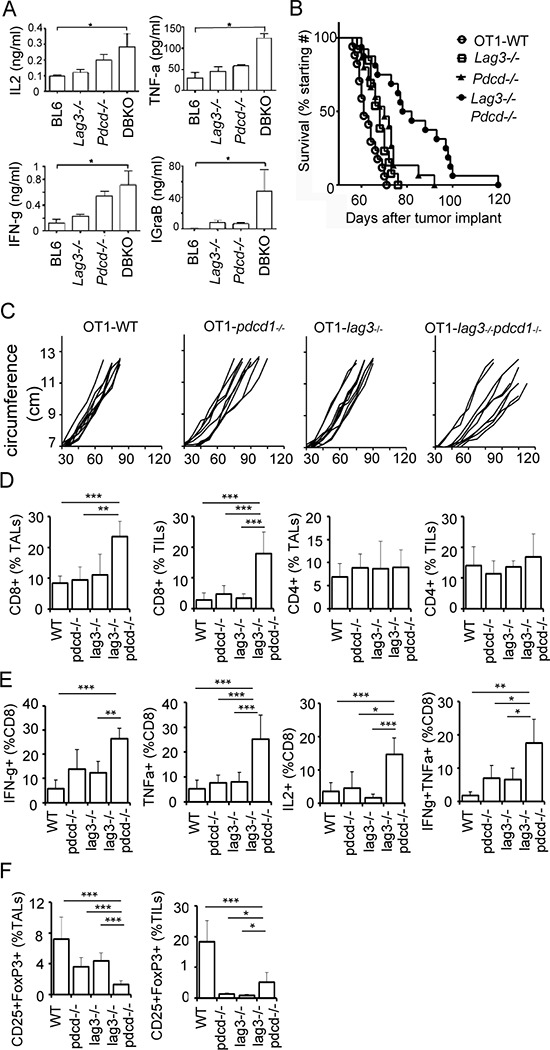
CD8^+^ T cells from Lag3^−/−^Pdcd1^−/−^ knockout mice exhibit enhanced effector phenotype **A.** Enhanced cytokine production by CD8^+^ T cells from *Lag3^−/−^*Pdcd1*^−/−^* mice. Purified naive mouse CD8^+^ T cells were isolated from C57BL/6, *Lag3^−/−^*, or *Pdcd1^−/−^*, or *Lag3^−/−^*Pdcd1*^−/−^* (DBKO) mice and activated with plate-bound anti-CD3/B7–1. Cytokines were measured from culture supernatants collected at 6 h or 24 h (*Granzym B*) post activation with ELISA. The statistics from three experiments were analyzed with one way ANOVA and error bars represent standard deviation between experiments. *P < 0.05. **B.** Enhanced anti-ovarian tumor immunity in OT-1-*Lag3^−/−^*Pdcd1*^−/−^* mice. IE9mp1 cells (1 × 10^7^ cells) were i.p. injected into wild-type OT-1 (OT1-WT, *n* = 17), OT1-*Lag3^−/−^* (*n* = 13), OT1-*Pdcd1^−/−^* (*N* = 15), and OT1-*Lag3^−/−^*Pdcd1*^−/−^* (*n* = 16) mice. Data are combined from repeated experiments, 3 to 5 animals per group. Data were analyzed using the Mantel-Cox log-rank test, *p* = 0.0001. **C.** Tumor growth monitored by measuring the abdominal circumvent surface. The ascitic fluid started accumulating at approximately 40 days in wild-type tumor-bearing mice post tumor implantation. Mice were euthanized when moribund or the circumference reached approximately 12 cm. **D.** Frequency of CD8^+^ and CD4^+^ TALs (ascites) and TILs (tumor) from tumor-bearing mice. TILs and TALs were isolated from late stage tumor-bearing mice (3–5 mice per group) and were prepared as described in the Material and Methods. Gating strategy is shown in Supplementary Figure 1. **E.** TILs and TALs from the *Lag3^−/−^*Pdcd1*^−/−^* mice contain significantly more cytokine producing cells upon SIINFEKL stimulation and **F.** Fewer CD25^+^ FoxP3^+^ cells. Error bars represent SD. Statistical significance was determined by Student's *t*-test. *P < 0.05; **p < 0.01; ***p < 0.001. Data shown are representative of 2 experiments.

Increasing evidence suggest that LAG3 and PD1 independently regulate autoimmunity [14, 15]; while simultaneous inhibition of LAG3 and PD1 enhances anti-tumor immunity or leads to autoimmunity [12, 16]. We have previously shown that human ovarian tumor-infiltrating CD8^+^ T cells co-expressing high levels of LAG3 and PD1 are dysfunctional and that dual blockade of both PD1 and LAG3 restores T effector function compared with blockade of either PD1 or LAG3 alone [10]. To test in a murine model whether inhibiting the PD1 and/or LAG3 pathway enhanced T effector function and antitumor immunity, we utilized wild-type C57BL/6, *Lag3^−/−^*, *Pdcd1^−/−^*, and *Lag3^−/−^*Pdcd1*^−/−^* double knockout mice. In order to use anti-OVA OT-1 T cells as a model, we also bred all the knockout mice into OT-1 background (H-2K^b^ restricted, anti-OVA TCR transgenic, on Rag2^−/−^ background) for the analysis of antigen-specific T cell responses. We first tested T cell effector function by analyzing the cytokine production by activated CD8^+^ T cells isolated from the *Lag3^−/−^*Pdcd1*^−/−^* mice and compared with those from wild-type (WT, C57BL/6) and the corresponding single knockout mice. During the course of a 24-h culture, CD8^+^ T cells derived from the *Lag3^−/−^* and *Pdcd1^−/−^* mice produced elevated levels of IL2, IFN-γ, TNF-α, and Granzyme B, as compared with those from the wild-type mice (Figure 1A). CD8^+^ T cells derived from *Lag3^−/−^*Pdcd1*^−/−^* double knockout mice produced even higher levels of all four cytokines than those from the single knockout mice. The results were most striking for Granzyme B where the levels exceeded the additive effects of inhibiting PD1 or LAG3 alone. To test whether single knockout *Lag3^−/−^* or *Pdcd1^−/−^* mice would reject ovarian cancer more efficiently than WT mice, mice (OT-1 background) were inoculated intraperitoneally with a highly aggressive and OVA-expressing mouse epithelial ovarian cancer line, IE9mp1. However, we observed only a small difference in survival among the animal groups (Figure 1B). These results indicated that inhibiting the PD1 or LAG3 pathway alone is not sufficient to control ovarian cancer. We then tested whether the two molecules synergize to affect CD8^+^ T cell immunity. Although a significant proportion of the BL6-*Lag3^−/−^*Pdcd1*^−/−^* lived for only 4–12 weeks due to severe autoimmune disease, the OT-1-*Lag3^−/−^*Pdcd1*^−/−^* lived 30–50% longer. We were able to challenge a small number of age matched mice (*n* = 16) that survived for long enough for the experiments. The data (Figure 1B) showed that OT-1-*Lag3^−/−^*Pdcd1*^−/−^* tumor-bearing mice exhibited significantly improved survival compared with OT-1-WT or single knock out OT-1-*Lag3^−/−^* or OT-1-*Pdcd1^−/−^* mice (*p* = 0.0001, Log-rank test). The tumor growth curves determined by the increased abdominal circumference resulting from the accumulation of ascitic fluid showed similar trend (Figure 1C). The findings that OT-1-*Lag3^−/−^*Pdcd1*^−/−^* mice control ovarian tumors better than the single knockout mice are consistent with previous reports in colon and melanoma models [27]. To investigate whether T cells contribute to the delay of tumor growth in the OT-1-*Lag3^−/−^*Pdcd1*^−/−^* mice, tumor infiltrating T cells (TILs) from the tumor bed and tumor associated T cells (TALs) from ascities were isolated from tumor bearing OT-1-*Lag3^−/−^*, OT-1-*Pdcd1^−/−^*, and OT-1-*Lag3^−/−^*Pdcd1*^−/−^* mice. The percentage of CD8^+^ TILs and TALs was significantly increased in the *Lag3^−/−^*Pdcd1*^−/−^* mice (Figure 1D; Supplementary Figure 1 for FACS gating). Importantly, TILs from the *Lag3^−/−^*Pdcd1*^−/−^* mice contained significantly more cytokine producing cells upon SIINFEKL peptide stimulation as compared with those from the single knockout mice. (Figure 1E; Supplementary Figure 2A for FACS gating). These TILs exhibited more poly-functionality since increased frequencies of IFN-γ ^+^TNF-α^+^-producing cells were observed (Figure 1E). The percentage of IFN-γ^+^IL2^+^ CD8^+^ TILs was not significantly different among the groups (data not shown). Although the percentage of CD4^+^ TILs and TALs were similar among different groups (Figure 1D), there were lower frequency of inhibitory CD25^+^ Fop3^+^ T regulatory (Treg) cells in the TILs from the OT-1-*Lag3^−/−^*Pdcd1*^−/−^* mice (Figure 1F). These data indicate that CD8^+^ T cells from OT-1-*Lag3^−/−^*Pdcd1*^−/−^* mice exhibit enhanced effector function and produce more inflammatory cytokines and suggest that LAG3 and PD1 synergistically promote immune tolerance in ovarian tumor bearing hosts.

### Combinatorial blockade of LAG3 and PD1 pathways enhance antitumor immunity in ovarian cancer

**Figure 2:**
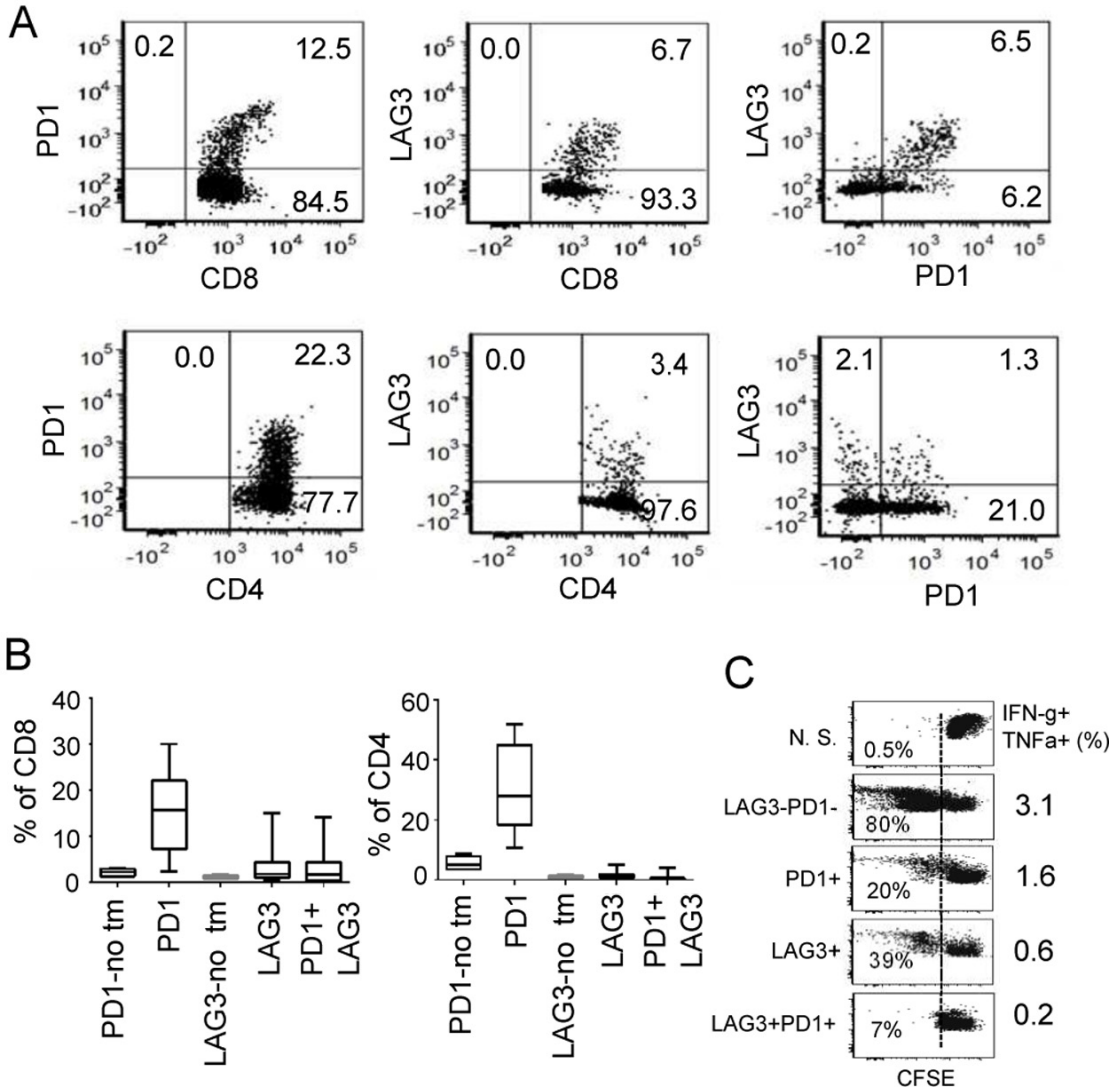
Co-expression of PD1 and LAG3 correlates with more severe dysfunction of CD8^+^ T cells **A.** Coexpression of LAG3 and PD1 in TILs from IE9mp1-tumor-bearing mice. Representative flow cytometry analysis of TILs stained with Live/Dead dye, mAbs to CD4, CD8, PD1 and LAG3. Dot plot analyses were gated on live cells, then on CD8 or CD4 and show percentages of single and double stained PD1- and LAG3-positive cells (see also Supplementary Figure 1). TILs were isolated from IE9mp1 tumors resected from wild-type (C57BL/6) mice 30–35 days post-implantation and stained for surface expression of the above mentioned protein and analyzed with flow cytometry. **B.** Pooled data of LAG3/PD1 expression on CD8^+^ or CD4^+^ TILs from wild-type (C57BL/6) mice. Data were obtained from 20 animals and analyzed as described in (A). **C.** Proliferation rate and function of PD1^+^, LAG3^+^ and PD1^+^LAG3^+^ TILs. CD8^+^ TILs from ascites and tumors were sorted by FACS or with CD8^+^ selection kit (Invitrogen), labeled with CFSE, and stimulated with plate-bound anti-CD3/CD28 antibodies for 4 days. CD8^+^ T cell proliferation was determined by dilution of CFSE; numbers in the FACS panels indicate the percentage of CFSE_low_ cells. Percentage of cells producing multiple cytokines (IFN- γ and TNF-α) is indicated in the panel to the right. Data shown are representative of three experiments.

**Figure 3:**
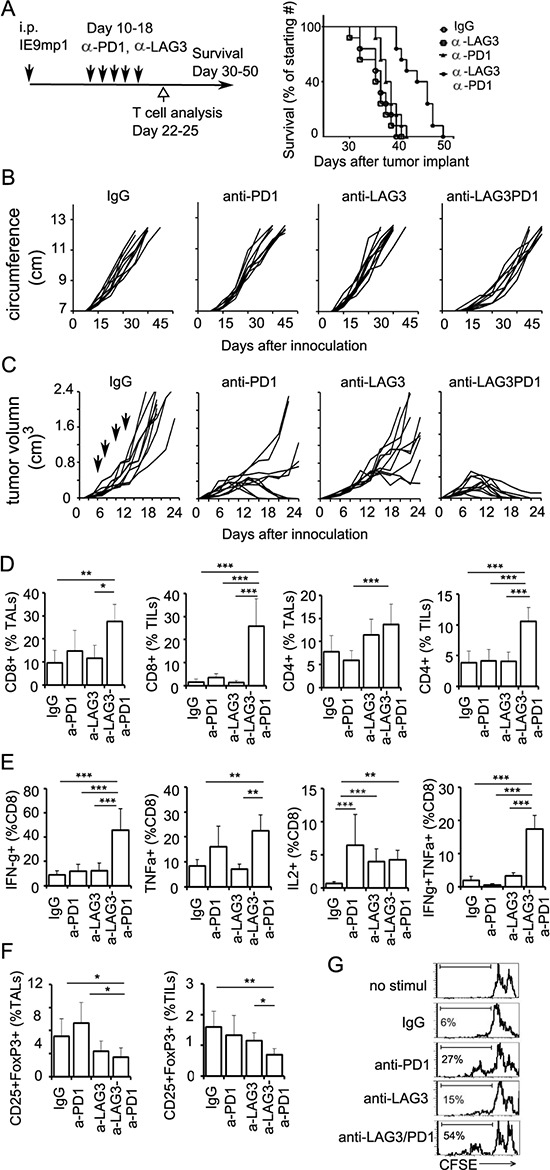
Dual blockade of LAG3 and PD1 synergistically enhance anti-tumor immunity **A.** Combinatorial anti-LAG3/anti-PD1 treatments delay ovarian tumor growth. Tumor-bearing mice C57BL/6 were randomized and treated with isotype control, anti-PD1, anti-LAG3, or anti-PD1/LAG3 combination on days 10, 12, 14, 16, and 18 (200 μg per treatment). Mice were euthanized when they developed ascites and their abdominal circumference reached 12 cm in diameter or when moribund. Data represent combined 10 mice per group. Data were analyzed with the Mantel-Cox log-rank test, *P* = 0.01. **B.** Tumor growth monitored by measuring the abdominal circumvent surface. The ascitic fluid started accumulating at approximately 20-25 days post tumor implantation. Mice were euthanized when moribund or the circumference reached approximately 12 cm. **C.** Combinatorial anti-LAG3/anti–PD1 treatment inhibits EG7 tumor growth in mice. C57BL/6 were randomized on day 6 when tumor volumes were approximately 40-60 mm^3^, and treated with isotype control, anti-PD1, anti-LAG3, or anti-PD1/LAG3 combination on days 6, 8, 10, and 12. Tumor volume was determined as described in Materials and Methods. Data shown are representative of 3 experiments with 10 mice per group. **D.** Frequency of CD8^+^ and CD4^+^ TALs (ascites) and TILs (tumor) from ovarian tumor-bearing mice post antibody blockade treatment. Both CD8^+^ and CD4^+^ TILs were significantly elevated in dual antibody-treated group. Mice were treated with 4 doses of antibodies on days 10, 12, 14, 16. TILs and TALs were isolated on day 22-25. **E.** Frequency of cytokine producing CD8^+^ TILs is enhanced after dual antibody blockade treatment. TILs or TALs were isolated as in (D) and stimulated with PMA/Ionomycin in the presence of BFA for 5 hours. Cytokine producing cells were analyzed as described in Material and Methods. Gating for flow cytometry analysis is shown in Supplementary Figure 2B. **F.** Dual antibody blockade treatment decreases CD25^+^FoxP3^+^ cells. Data are representative of 3 independent experiments with 3-5 animals per group. Error bars represent SD. Statistical significance was determined by Student's *t*-test. *P < 0.05; **p < 0.01; ***p < 0.001. **G.** Dual antibody blockade enhances the proliferation rate of CD8^+^ TILs. TILs were pulled from 2 mice and sorted by CD8^+^ selection kit (Invitrogen or STEM cell technology) and CFSE dilution assay was performed as described in Figure 2C. Percentage of CD8^+^ T cells with diluted CFSE label is shown. Data shown are representative of three experiments.

To further demonstrate the potential collaboration between LAG3 and PD1 in promoting T cell tolerance in the tumor microenvironment, we asked whether PD1 and LAG3 are co-expressed on tumor infiltrating T lymphocytes (TILs) of murine ovarian cancer in C57BL/6 mice. We observed that within TILs, approximately 15% of the CD8^+^ and 24% of the CD4^+^ T cells expressed PD1 (Figure 2A–2B, Supplementary Figure 1 for FACS gating). Approximately 2–10% of these TILs also expressed LAG3. Interestingly, 90% of these LAG3-epressing CD8^+^ T cells co-expressed PD1. In contrast, splenocytes of non-tumor bearing mice contained approximately 2–5% of CD8 and CD4 cells that expressed PD1; and 1–3% that expressed LAG3 (Figure 2B, PD1-no tm and LAG3-no tm). Similarly, only 2–5% of the CD8 and CD4 cells from the spleens of the mice bearing IE9mp1-tumors expressed PD1 or LAG3 (data not shown). The PD1^+^LAG3^+^ TILs are hypo-responsive upon TCR stimulation, as indicated by the decreased proliferation rate and percentage of cells that produced both IFN-γ and TNF-α (Figure 2C). Although some of the PD1^+^LAG3^+^ TILs were single cytokine producing cells (Supplementary Figure 2B), almost none of them produced both IFN-γ and IL2 (data not shown). These results are consistent with the findings in human EOC TILs [10], suggesting that co-expression of PD1 and LAG3 on T cells in tumor microenvironment is associated with T-cell exhaustion. Previous reports have shown enhanced anti-tumor activity by combinatorial treatment with both anti-LAG3 and anti-PD1 antibodies in murine colon cancer but not in melanoma [12]. We evaluated whether dual antibody blockade of both LAG3 and PD1 pathways would reduce the IE9mp1 ovarian tumor growth in C57BL/6 (WT) mice. The IE9mp1-tumor bearing C57BL/6 mice started generating visible ascites around day 20. Antibody blockade was performed from day 10-18 (5 doses at 200 μg per treatment, delivered every 2 days). Consistent with the results of the tumor-bearing *Lag3^−/−^* and *Pdcd1^−/−^* OT-1 mice (Figure 1A), treating the C57BL/6 mice with single agent blockade did not have a clear benefit, however, combinatorial blockade with anti-LAG3 and anti-PD1 antibodies significantly delayed the growth of the IE9mp1 ovarian tumors (Figure 3A, *p* = 0.01). Tumor growth was also determined by measuring the increased accumulation of abdominal ascitic fluid (Figure 3B). To further confirm the effects of dual anti-LAG3 and anti-PD1 blockade in delaying tumor growth using an additional tumor model, we further tested antibody blockade in the context of the subcutaneous OVA-expressing lymphoma EG7 tumor. Remarkably, treatment with dual anti-LAG3/PD1 antibodies (4 doses at 200 μg per mouse, delivered every other day) resulted in tumor rejection in 100% of the mice (Figure 3C). Treatment with single agent anti-PD1 antibody also promoted 50% of tumor rejection while anti-LAG3 blockade treatment significantly delayed tumor growth as well when compared with the IgG control treatment. The roles of T cells in mediating the delay of IE9mp1 tumor growth were analyzed. The percentage of both CD8^+^ and CD4^+^ TILs and TALs was significantly increased after dual anti-LAG3 and anti-PD1 antibody blockade as compared with treatment with the IgG control and single antibody (Figure 3D). Importantly, dual blockade significantly increased the effector function of the CD8^+^ TILs as judged by the increased frequency of single- and dual-cytokine (IFN-γ - TNF-α) producing CD8^+^ TILs (Figure 3E) and higher rate of proliferation (Figure 3G). The frequency of cytokine-producing TALs showed similar trend (data not shown). In addition, treatment with dual anti-LAG3/PD1 antibodies significantly decreased the frequency of inhibitory CD25^+^ Fop3^+^ Treg cells (Figure 3F). Together, these results indicate that dual LAG3 and PD1 blockade enhance antitumor immunity in the ovarian tumor microenvironment through increases T cell infiltration, elevated T effector function as well as reduction in infiltration by immunosuppressive Tregs.

### Co-expression and co-localization of LAG3 and PD1 in OT1 CD8^+^ T cells at various cellular compartments

**Figure 4:**
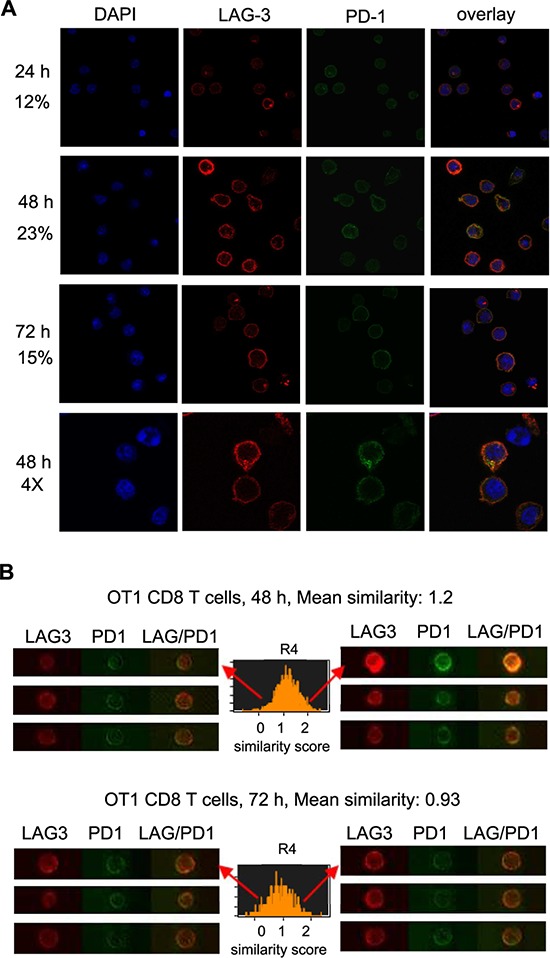
Co-expression and co-localization of LAG3 and PD1 during T cell activation **A.** LAG3 co-localized with PD1 during CD8^+^ T cell activation peaked at 48 h post activation. CD8^+^ T cells freshly isolated from OT-1 mice were activated with plate-bound anti-CD3/B7.1 for various times (24 h, 48 h, 72 h). Cells were stained for LAG3 (Cy3, red) and for PD1 (AlexaFluor 488; green), and analyzed using confocal microscopy. DAPI (blue) was used as counterstain for the nucleus. Examples of single-plane confocal images are shown. The percentage of T cells with LAG3 and PD1 co-localization was quantified by counting the cells with yellow signals derived from the merge of the red and green images in the overlays (*N* = 300) using the color picker tool in the ImageJ program and is indicated on the left of each panel. Data shown are representative of three independent experiments. **B.** LAG3 and PD1 expression and co-localization using ImageStream at 48 h, and 72 h after activation. The co-localization of LAG3 and PD1 was examined by looking at the similarity score (coincidence of both signals) determined by the software's algorithms. A histogram is plotted showing the similarity scores (R4) for the entire population, and a mean score calculated. The more to the right the curve is the more co-localized the proteins. Data shown are representative of two experiments.

To investigate the possibility that LAG3 and PD1 interact to exert their co-inhibitory effect on T cells, we analyzed the temporal and spatial co-expression of these proteins in activated CD8^+^ T cells from OT-1 mice. First, using flow cytometry analysis, LAG3 and PD1 were found to be co-expressed on the CD8^+^ T cell surface at 24 h following activation in approximately 24% of cells and the expression peaked at 48-72 h post activation in approximately 90% of cells (data not shown). Next, to assess their subcellular location we visualized the permeabilized CD8^+^ T cells using confocal microscopy. While LAG3 and PD1 were clearly expressed on the cell surface, there appeared to be some LAG3 and PD1 in the T-cell cytoplasm (Figure 4A). Interestingly, we found that LAG3 and PD1 co-localization peaked at 48 h post activation as estimated by the intensity of the signals and counting the percentage of cells with overlapping signals of red (LAG3) and green (PD1). For quantitative measurement of LAG3 and PD1 co-expression and localization, activated OT-1 CD8^+^ T cells were analyzed with ImageStream multispectral imaging flow cytometer as illustrated in Figure 4B. The mean similarity scores indicate that the frequency of PD1 and LAG3 co-localization is higher at 48 h (mean similarity score, 1.2) than at 72 h (mean similarity score, 0.93) post activation. The data confirmed the observation made using confocal microscopy (Figure 4A) that the expression of LAG3 and PD1 increase over time and they co-localized in approximately 15% of the population at 48 h post activation.

**Figure 5:**
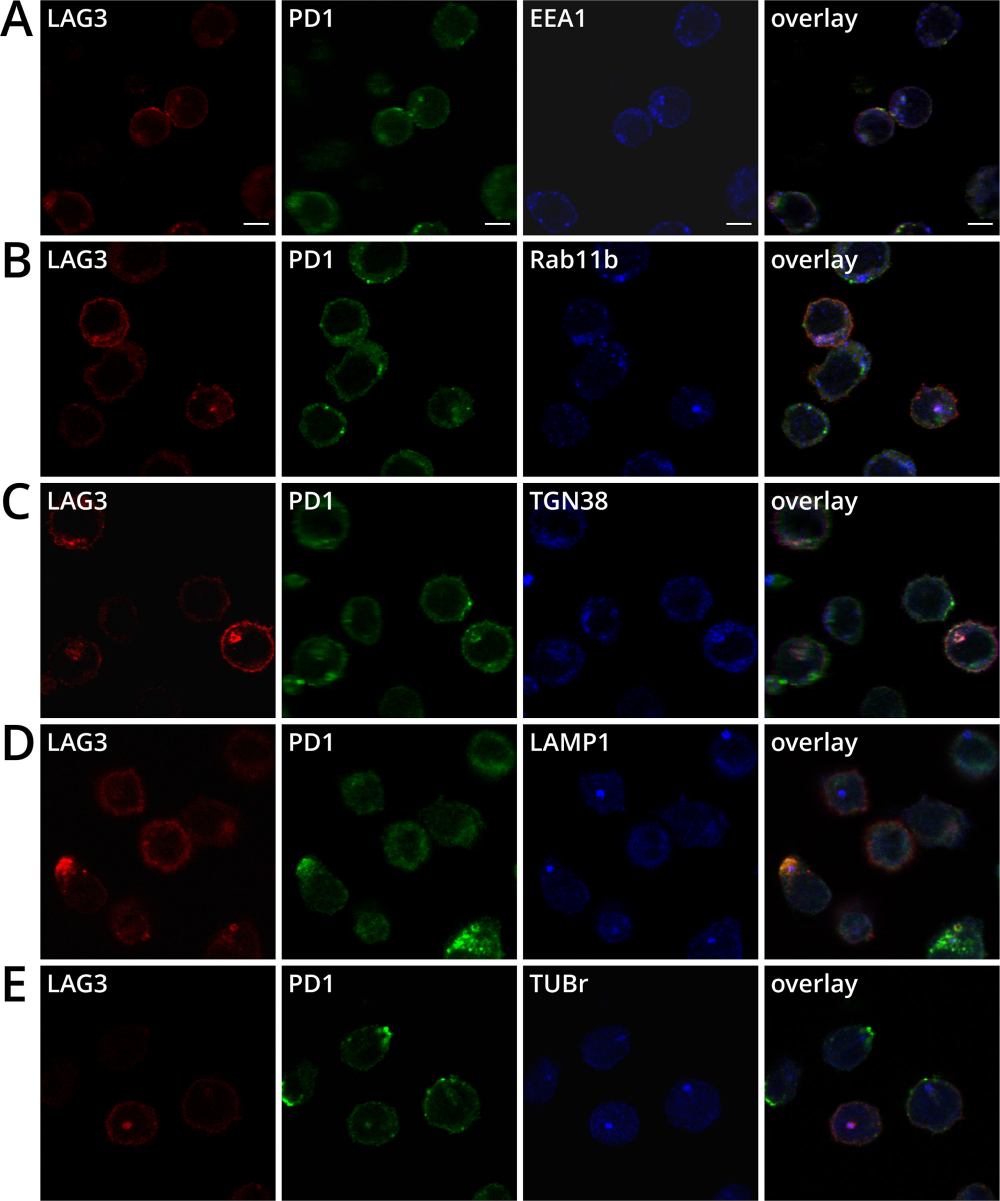
Sub-cellular co-localization of LAG3 and PD1 in cytoplasmic compartments in activated CD8^+^ T cells OT-1 T cells were activated and prepared as described in Figure 4A and stained with anti-LAG3 (CY3, red), anti-PD1 (AlexaFluor488, green), plus either **A.** anti-EEA1 (AlexaFluor647, blue) or **B.** anti-Rab11b (AlexaFluor647, blue), **C.** anti-TGN38 (DyLight649, blue), **D.** anti-LAMP1 (AlexaFluor647, blue), **E.** anti-tubulin- γ (AlexaFluor647, blue) and analyzed with confocal microscopy. Single plane images are representative of two independent experiments. Scale bar represents 5 μm. Co-localization is demonstrated by the white color derived from the merge of the red, green and blue channels. The percentage of T cells with LAG3 and PD1 co-localizing with the cellular compartments (*N* = 300) was performed as described in Figure 4A.

The intracellular localization of LAG3 or PD1 has been previously shown separately [27, 28]. It was reported that approximately half of LAG3 molecules are retained intracellularly and appear to reside close to the early and recycling endosomes, the secretory lysosomes, and the microtubule organizing center (MTOC) [27]. PD1 has also been shown to localize near the Golgi matrix protein GM130 and the trans-Golgi network (TGN) protein TGN38, but not the secretory lysosomes [28]. To further assess the sub-cellular co-localization of LAG3 and PD1, we used antibodies against several markers for these cytoplasmic compartments. Both LAG3 and PD1 were found co-localized in some cells with the early and recycling endosomes using early endosomal antigen 1 (EEA1) and Rab11b as markers, respectively, (Figure 5A–5B). These markers may represent newly synthesized proteins that are on route to the plasma membrane and/or in the process of recycling. In addition, LAG3 and PD1 also co-resided with TGN38 (Figure 5C) and secretory lysosome marker LAMP-1 (Figure 5D). Similar to previous findings [27], LAG3 appeared to be strongly co-localized with the MTOC, using γ-tubulin as a marker (Tub-r, Figure 5E); similar frequency of PD1 and MTOC co-localization was observed. Overall LAG3 and PD1 co-localize with MTOC in approximately 14% of the activated cells.

**Figure 6:**
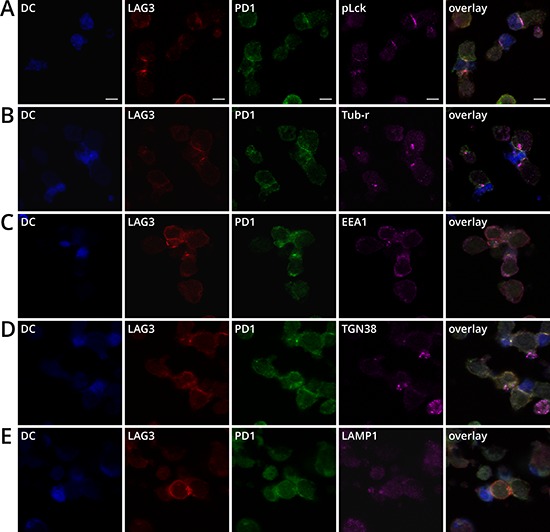
LAG3 and PD1 co-localization with pLCK, MTOC, EEA1, TGN38, and LAMP1 near the immunological synapse **A.** LAG3 (red) and PD1 (green) concentrated at the synapse of conjugates between activated DCs and OT1 CD8^+^T cells. Splenic DCs from BL6 mice were isolated with CD11c^+^ beads and activated with GMCSF and LPS and pulsed with SIINFEKL for 20 h. DCs stained with Cell Tracker Blue (CTB) were mixed with activated T cells for 20 min to form conjugates. Conjugates with concentrated pLck (magenta) at the synapse were scored for the co-localization. **B.** Microtubule organization center (MTOC) polarized toward the synapse. Tubulin- γ (magenta) was used as a marker for MTOC. **C.** Clustering of EEA1, and **D.** TGN38, and **E.** LAMP1 near the LAG3 (red) and PD1 (green) co-localization at the synapse.

The overlapping intracellular location of LAG3 and PD1 at the proximity of vesicle trafficking compartments suggests that LAG3 and PD1 may be stored closely in the same intracellular compartments and travel together to plasma membrane, possibly through MTOC, to exert their inhibitory effects during T cell signaling. It has been previously demonstrated that PD1 accumulates extensively at the immunological synapse (IS, defined as enrichment at the T cell and antigen presenting cell contact site) when T cells interact with dendritic cells (DCs) expressing high levels of B7-DC [29]. LAG3 has also been shown to be present in the IS [30]. We thus examined whether LAG3 and PD1 also co-localized at the immunological synapse. Activated OT-1 cells were incubated with LPS-matured DCs that were pulsed with SIINFEKL to form conjugates. We used phospohorylated Lck (pLck) as a marker for the IS since Lck of the Src-family protein kinases has been shown to cluster at the supramolecular activation clusters [31]. The distribution of LAG3, PD1, and pLck was scored with respect to the IS. Indeed, LAG3 and PD1 were found co-localized at the immunological synapse in most of the T cell-DC conjugates (Figure 6A). It is well known that MTOC reorients toward the IS [32] and mediates trafficking of polarized vesicles to the IS [33]. Indeed, Figure 6B shows that the MTOCs in both T cells and dendritic cells polarized toward the IS and near the area of LAG3 and PD1 colocalization. In addition, the endosomal (EEA1) trans-Golgi network (TGN38), and lysosomal markers (LAMP-1) in the T cells also increasingly accumulated near the IS where LAG3 and PD1 concentrated (Figure 6C–6E). Under this condition, trans-Golgi network and MTOCs were found near the IS in 50% and 75%, respectively, of the T cell-DC conjugates, while EEA1 and LAMP1 were found in approximately 30-40%. Together these data revealed that LAG3 and PD1 shared partially overlapping trafficking pathways and traveled to the IS, likely through the MTOC, and concentrated at the IS formed between T cells and antigen-presenting cells.

### LAG3 interacts with PD1 during CD8^+^ T cell signaling

**Figure 7:**
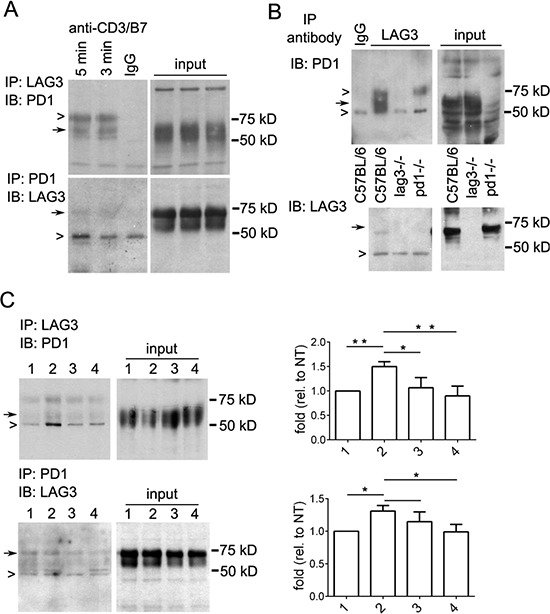
Immunoprecipitation reveals interaction between LAG3 and PD1 in restimulated T cells **A.** LAG3 and PD1 association in rested and restimulated CD8^+^ T cells. Activated OT1 cells were rested in IL2 for 3 days and restimulated (2 × 10^7^) with plate-bound anti-CD3/B7.1 for 3 or 5 min before lysis. Lysates were immunoprecipitaed with anti-LAG3 (upper panels or anti-PD1 (lower panels) antibodies or control IgG (lane 3, IgG). Immunoprecipitated complexes were detected by Western blotting first using a different anti-PD1 antibody (raised in goat, upper panel) or anti-LAG3 antibody (lower panel). Closed arrows indicate specific bands corresponding to LAG3 or PD1; open arrows represent IgG or nonspecific bands. Input extract (~0.5% of total lysate) was used as markers and loading control. **B.** LAG3 association with PD1 was abolished in CD8^+^ T cells isolated from *Lag3^−/−^* or *Pdcd1^−/−^* mice. CD8^+^ T cells were isolated from C57BL/6, *Lag3^−/−^*, and *pdcd1^−/−^* mice using CD8^+^-Dynal beads, activated with plate bound anti-CD3/B7.1 for 48 h, expanded with IL2, restimulated with anti-CD3/B7.1. Immunoprecipitation and Western blotting were performed as described in (A). **C.** LAG3 and PD1 association was reduced by blocking anti-PD-L1 and anti-PD1 antibodies. Activated OT1 cells were rested in IL2 for 3 days and re-stimulated (2 × 10^7^) with OVA- and PD-L1-expressing tumor cells (IE9mp1, 3 × 10^5^) for 3 (lane 1) or 5 minutes (lane 2–4). IE9mp1 cells (lane 3) or both OT1 and IE9mp1 cells (lane 4) were pretreated with blocking anti-PD-L1 (20 μg/ml) and anti-PD1 (20 μg/ml) antibodies for 2 h before restimulation and lysis. Immunoprecipitation and Western blotting were performed as described above. Data shown are representatives of three independent experiments. Densitometry measurement of the bands was performed using ImageJ. The amount of PD1 (top) or LAG3 (bottom) pulled-down by each other was normalized with that of the input and shown on the panels to the right. Error bars represent SD. *p < 0.05; **p < 0.01

To further define the interaction between LAG3 and PD1 during CD8^+^ T cell signaling, reciprocal immunoprecipitation with specific antibodies for LAG3 and PD1 was used. Herein for *in vitro* experiments, the primary OT-1 CD8^+^ T cells were activated for 48 hours and rested in IL2 for 3 days. These cells after resting for 3 days had a semi-exhausted phenotype since they expressed lower level of CD69 and produced lower cytokine as compared with the activated T cells at 48-72 hour post stimulation (Supplementary Figure 3). The lower level of PD1 in comparison with LAG3 was possibly due to epigenetic down regulation of PD1 post activation [34]. For detecting T cell signaling, these cells were re-stimulated with anti-CD3/B7.1 for 3 or 5 minutes. As shown in Figure 7A, a broad band of protein about 50-55 kDa corresponding to PD1 was co-immunoprecipitated by anti-LAG3 antibody (top panel). Similarly, a band of about 70 kDa corresponding to full length LAG3 is co-immunoprecipitated by anti-PD1 antibody (Figure 7A bottom panel). It appeared that the 54 kDa protein corresponding to the LAG3-lacking the cytoplasmic domain was very weak or not present in the LAG3 and PD1 immunoprecipitates, possibly due to its low level in the input. Since LAG3 and PD1 colocolize in 10-15% of the activated CD8 T cells (Figure 4), we reasoned that the LAG3-PD1interacting complex maybe transient and weak. To further demonstrate the specificity of the interaction, immunoprecipitates of CD8^+^ T cells from C57BL/6 and *Lag3^−/−^* and *Pdcd1^−/−^* mice were compared. As shown in Figure 7B, LAG3 and PD1 immunoprecipitates were present only in the CD8^+^ T cells from wild-type C57BL/6 mice but not those from *Lag3^−/−^* or *Pdcd1^−/−^* mice, respectively. The association of LAG3 and PD1 also occurred in OT-1 CD8^+^ T cells that were re-stimulated with antigen (OVA)-expressing tumor cells (IE9mp1) (Figure 7C, lane 1 and 2). The IE9mp1 cells used here were also treated with IFN-γ to induce PD-L1 expression (data not shown) to serve as the source of PD-L1 for PD1 ligation. The interaction of PD-1 with LAG-3 was significantly reduced (30-40%) by treatment of the tumor and T cells with blocking antibodies anti-PD-L1 and anti-PD1, respectively (Figure 7C, lane 4), while treatment with anti-PD-L1 on tumor cells alone had a lesser effect (Figure 7C, lane 3). Thus, our data revealed evidence of interaction between LAG3 and PD1 during CD8^+^ T cell activation which was partially dependent on PD-1 ligation.

### LAG3 is associated with PD1-SHP1/SHP2 interacting complex

**Figure 8:**
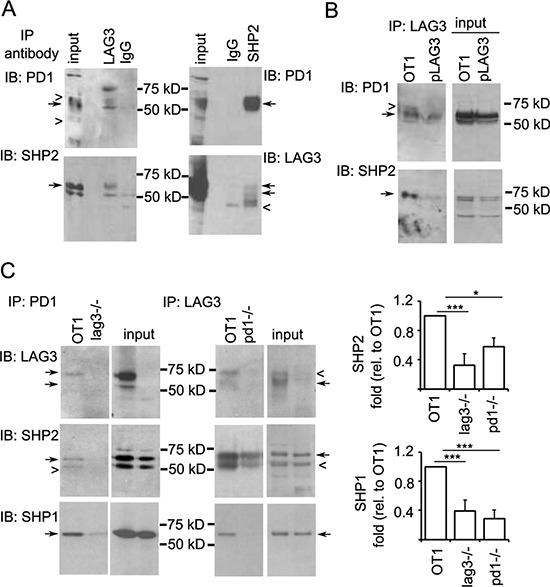
LAG3 associates with PD1-SHP2 or -SHP1 interacting complex **A.** Reciprocal immunoprecipitation of LAG3 and SHP2. A LAG3 antibody pulled down PD1 and SHP2 (left panels) and a SHP2 antibody pulled down PD1 and LAG3 (right panels). OT-1 T cells were activated with SIINFEKL or anti-CD3/B7.1 for 2 days and expanded with IL2 for 3 days. Rested cells (2 × 10^7^) were washed and treated with pervanadate for 5 mins, lysed and immuno-precipitated with anti-LAG3- or anti-SHP2- or control IgG-conjugated Dynal beads. Western blots were probed sequentially with anti-PD1 and anti-SHP2 (left panels) or anti-LAG3 (right panels) antibodies. **B.** LAG3 association with SHP2 is confirmed in the *Lag3^−/−^* CD8^+^ T cells reintroduced with LAG3. Activated CD8^+^ T cells from *Lag3^−/−^* mice were transduced with a retrovirus vector expressing LAG3 (pLAG3), expanded with IL2 and stimulated with pervanadate for 5 min, lysed and immunoprecipitated with anti-LAG3-conjugated Dynal beads. Western blots were probed sequentially with anti-PD1 and anti-SHP2 antibodies. OT1 CD8^+^ T cells were used as a positive control. **C.** Reduced recruitment of SHP2 and SHP1 in *Pdcd1^−/−^* and *Lag3^−/−^* CD8^+^ T cells. Activated CD8^+^ T cells were rested in IL2 and restimulated with OVA- and PD-L1-expressing tumor cells (IFN-γ-treated IE9mp1) for 5 min. Lysates were prepared, immunoprecipitated, and analyzed as described in Figure 7C. Data shown are representative of two independent experiments. Quantitation of the amount of SHP2 (middle panels) and SHP1 (bottom panels) pulled-down by anti-PD1 or anti-LAG3 antibody was normalized with that of the input and shown on the panels to the right. Error bars represent SD. *p < 0.05; ***p < 0.001

The data described above that LAG3 and PD1 co-localize with phosphorylated Lck at the immunological synapse suggest that LAG3 and PD1 may collaborate in orchestrating their inhibitory effect on T cell signaling. While SHP2 has been shown in multiple studies to be recruited by PD1 to inhibit TCR signaling [19, 35, 36], SHP1 has only been shown to be recruited by PD1 in primary human T cells and with much lower affinity to PD1 than SHP2 [36]. We investigated whether LAG3 protein is present in the PD1-SHP1 or SHP2 interacting complex. For this purpose, OT-1 cells were activated with anti-CD3/B7.1 (or SIINFEKL) and expanded. Since PD1 phosphorylation is required for the recruitment of SHP1 and SHP2 [35, 37], the rested CD8^+^ T cells were treated with pervanadate as previously described [36]; this leads to maximal phosphorylation of tyrosine residues before lysis. As shown in Figure 8A, PD1 and SHP2 were both present in the anti-LAG3 precipitates (close arrows). In addition, reciprocal immunoprecipitation with anti-SHP2 antibody pulled down both PD1 and LAG3, although the LAG3 signal was weak. These results were confirmed using CD8^+^ T cells from *lag3 ^−/−^* mice that were transduced with a plasmid expressing wild-type LAG3 (Figure 8B, pLAG3). These data indicate that LAG3 is present in the PD1-SHP1/2 complex during T cell signaling. To test whether the association of LAG3 with PD1 results in enhanced recruitment of SHP1/SHP2 that could explain increased attenuation of T cell signaling, we stimulated LAG-3 deficient T cells with antigen. Indeed, when stimulated with IE9mp1 ovarian tumor cells, less SHP1 and SHP2 were pulled-down by anti-PD1 antibody in the *Lag3^−/−^* cells (Figure 8C, left panels). Interestingly, less SHP1 and SHP2 were pulled-down by anti-LAG3 antibody in *Pdcd1^−/−^* cells as well (Figure 8C, right panels). These data suggest that LAG3 may help the recruitment of SHP1/SHP2 by PD1 but LAG3 may also be associated with a different phosphatase-recruiting complex in the absence of PD1.

### The ITIM and ITSM of PD1 is necessary while the cytoplasmic domain of LAG3 is not absolutely required for LAG3 and PD1 interaction

**Figure 9:**
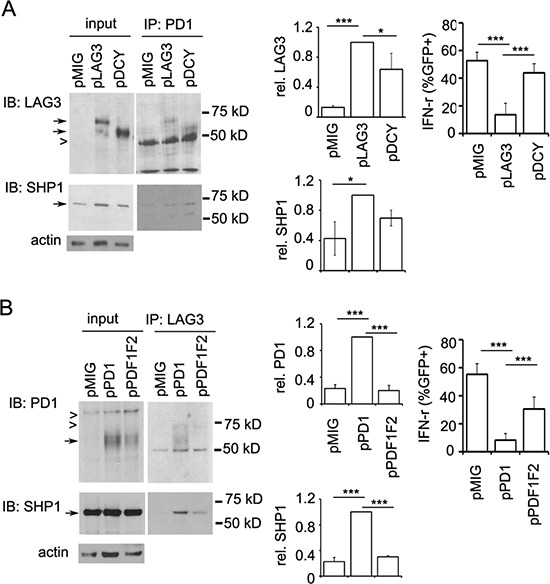
Differential contribution of the cytoplasmic domains of LAG3 and PD1 in their interaction **A.** LAG3 protein lacking cytoplasmic domain associates with PD1 with slightly reduced efficiency. Wild-type LAG3 (pLAG3) or LAG3 protein lacking the cytoplasmic domain (pΔCY), or pMIG vector control constructs were transduced into activated *Lag3^−/−^* CD8^+^ T cells. Sorted GFP^+^ cells were rested with IL2 for 2 days and restimulated with IE9mp1 for 5 min before lysis. Lysates were immunoprecipitated with anti-PD1 antibody and probe with anti-LAG3 (top panels) and then with SHP1 (lower panels) as described above. Densitometry analysis of the corresponding bands to calculate the ratio of pLAG3 versus pDCY was performed using the ImageJ software. Relative luminescence units were normalized with input levels and expressed as a ratio relative to pLAG3. Data shown are representative of three (LAG3) or two (SHP1) independent experiments. **B.** Mutations in the ITIM and ITSM of PD1 compromise its association with LAG3. Wild-type PD1 (pPD1) or mutant PD1with mutations in the ITIM and ITSM (pPDF1F2) or control pMIG vector constructs were transduced into activated *Pdcd1^−/−^* CD8^+^ T cells. Transductants were sorted and immunoprecipitation performed as described above. Data shown are representative of two independent experiments. Relative luminescence units were normalized with input levels and expressed as a ratio relative to pPD1. Error bars represent SD. *p < 0.05; ***p < 0.001.

Next, we investigated whether the association of LAG3 and PD1 is mediated through their cytoplasmic domains which have both been shown to be important for T cell signaling and function. LAG-3 is cleaved within the connecting peptide (CP) between the D4 Ig domain and the transmembrane domain into two membrane-associated fragments [22]: a 54-kDa fragment that contains all the extracellular domains and a 16-kDa peptide that contains the transmembrane and cytoplasmic domains. Thus we first ectopically expressed the full length LAG3-WT-GFP (pLAG3) or the 54-kDa fragment-containing LAG3 (pΔCY) along with the vector control (pMIG) in CD8^+^ T cells derived from *Lag3^−/−^* mice. Co-immunoprecipitation assay with anti-PD1 antibody clearly showed that, similar to wild-type LAG3, the “cytoplasmic-less” LAG3 (ΔCY) was pulled down by anti-PD1 antibody, albeit with reduced level (Figure 9A top panels). Similarly, the level of SHP1 pulled down by anti-PD1 antibody was mildly reduced in CD8^+^ T cells expressing the cytoplasmic-less LAG3 construct (Figure 9A, bottom panels). Quantitation using densitometry measurement indicated that the amount of pΔCY pulled down by anti-PD1 antibody was ~30% lower than that of the pLAG3, and the amount of SHP1 pulled down by anti-PD1 antibody in the presence of pΔCY was 20% less than that of pLAG3. Similar data were obtained for the SHP2 pulled down by the anti-PD1 and anit-LAG3 antibodies. These results indicate that the cytoplasmic domain of LAG3 is not critical for LAG3-PD1 association, although the association is attenuated in its absence. In contrast, functional studies indicated that the cytoplasmic-less LAG3 abolished 80% of the inhibitory effect of LAG3 on IFN-γ production (Figure 9A, top right panel). These data suggest that while the extracellular domain of LAG3 can associate with PD1, it is the cytoplasmic domain that accounts for most of the inhibitory function of LAG3 when it associates with PD1. The requirement of the ITIM and ITSM of PD1 in LAG3-PD1 association was also tested by ectopically expressing the wild-type PD1 (pPD1 in Figure 9B) or the mutant carrying mutation on both ITIM and ITSM (pPDF1F2) along with the vector control (pMIG) in CD8^+^ T cells derived from *pdcd1^−/−^*mice. While PD1 was clearly pulled down by anti-LAG3 (Figure 9B, top panel), PDF1F2 level was significantly reduced. Similarly, recruitment of SHP1 was significantly reduced in the pPDF1F2-expressing cells as compared with that in the PD1-expressing cells. Similar to previous report [35], the ITIM and ITSM of PD1 is essential for the PD1-mediated inhibitory effect on T cells function as indicted by the reversal of PD1-mediated inhibition of IFN- γ production (Figure 9B, right panel). Collectively, our data suggest that the cytoplasmic domain of LAG3 plays a minor role but the ITIM and ITSM of PD1 are necessary for PD1and LAG3 association and that they are both important in regulating CD8^+^ T cell function.

## DISCUSSION

In this report we set out to understand the possible molecular mechanisms by which LAG3 and PD1 co-regulate T cell function during immunity and tolerance. Our results demonstrate the following: (i) LAG3 and PD1 are elevated and co-expressed in TILs from murine ovarian tumor- and EG7-bearing mice and dual blockade of both molecules enhances antitumor immunity via enhanced CD8^+^ T effector frequency and function and reduced frequency of Treg cells in the tumor microenvironment. (ii) LAG3 and PD1 are co-expressed both spatially and temporally in activated T cells and share partially overlapping cell surface and intracellular locations and trafficking pathways. (iii) Both LAG3 and PD1 co-localized with phosphorylated Lck at the immunological synapse formed between the CD8^+^ T cells and DCs conjugates. (iv) The co-localization of LAG3 and PD1 occurs in vesicle trafficking compartments including MTOC, EEA1, TGN38, and LAMP1, and are enhanced at the IS. (v) LAG3 interacts with PD1 during CD8^+^ T cell signaling and plays a role in recruiting SHP1/2 to attenuate T cell signaling and exert their co-inhibitory regulatory roles.

The enhanced co-localization of LAG3 and PD1 with both endosomal and Golgi network and near the MTOC at the immunological synapse (IS) supports the idea that LAG3 and PD1 are rapidly transported and recycled to the plasma membrane via the same route. At the IS of T cell and APC, both LAG3/MHC class II interaction and PD1/PDL-1 ligation associate with the CD3/TCR complex [38]; and can each exert negative signals independently. However, together they have synergistic or additive inhibitory effects as demonstrated by the (i) increased amount of inflammatory cytokines/granzyme B secreted by the CD8^+^ T cells from the double knockout mice as compared with the single knockout cells, (ii) changes in the recruitment of SHP1 and SHP2 in the single knockout mice, (iii) enhanced autoimmunity in the double knockout mice [16] and elevated antitumor activity by the combinatorial antibody blockade (Figure 3, [12]). In contrast to our finding here, others have found that a triple combinatorial blockade with anti-CTLA4/PD-1/LAG3 antibodies have no effect on a different ovarian tumor model [39]. We suspect that the discrepancy may be due to the differences in the doses and schedules of the antibodies administered and the cell lines used.

A limitation of our immunoprecipitation data is that the approach may not capture the full magnitude of the LAG3 and PD1 interaction, but only a transient association of the two molecules. As the immunoprecipitation signals were not strong, it is plausible that they interact indirectly as part of a complex or they may be tethered to cytoskeletal elements in the same compartment. The data showing co-localization of LAG3 and PD1 with the cytoplasmic compartments and near the MTOC (Figure 5) would support this possibility. It is also possible that both LAG3 and PD1 interact with other molecules at the same time given the fact that there exist other inhibitory receptors in T cells. Alternatively, LAG3 is known to form homo-dimers and oligomers [25] which will leave only a window of opportunity for it to associate with other molecules. However, this seems to be unlikely since LAG3 and PD1 co-localize readily and strongly at the IS in most of the T cell-DC conjugates (Figure 6A). Future studies using FRET (Fluorescence resonance energy transfer) technique in tumor infiltrating CD8^+^ T cells will help to determine whether and where these molecules directly interact and whether the colocalization of LAG3 and PD1 in TILs correlates with T cell dysfunction.

Our data also reveals that LAG3 is present in the PD1 and SHP1 or SHP2 interaction complex. Since the amount of LAG3 in the anti-SHP2 antibody pull down assay is not strong, LAG3 may not directly interact with the phosphatases. Phosphatases such as SHP-1, SHP-2, or SHIP have been reported to be recruited by ITIM-bearing immunoreceptors, one of which is PD1, and are critical for their inhibitory function. LAG3 does not contain an ITIM or the ITSM. This will suggest that the cell-intrinsic negative regulatory function of LAG3 is mediated via the interaction of PD1 and the phosphatases. However, it is also plausible that LAG3 interacts with adaptor proteins, such as Grb2, which has been shown to bind to SHP2 [40] and whose SH3 domains can directly complex with proline-rich region of other proteins [41]. Furthermore, our observation that LAG3 and PD1 are closely associated with MTOC in activated T cells and at the immunological synapse strongly suggests that LAG3 and an unidentified protein, possibly similar to the LAG3-associated protein (LAP) which interacts with the EP region of LAG3 in human T cells, may act as docking proteins for PD1 and facilitates translocation of these proteins via MTOC to the plasma membrane to exert their negative signaling functions. The importance of the EP region in the translocation of LAG3 to cell surface has also been recently demonstrated [26].

Our limited mutational analysis suggested that the association of LAG3 with PD1 does not completely involve the cytoplasmic domain of LAG3 whereas it requires the ITIM and ITSM of PD1. Both LAG3 and PD1 have multiple functional domains. While our data indicate that LAG3 without the cytoplasmic domain is able to interact with PD1 with slightly reduced efficiency, this domain clearly contributes significantly to inhibiting T cell function (IFN- γ production). The later finding is in consistent with a previous report that a conserved KIEELE motif in the cytoplasmic domain of LAG3 contributes to the inhibitory effect of LAG3 on CD4-dependent T cell function [42]. We also observed that LAG3-deficient T cells carrying the pΔCY construct do not expand *in vitro* as well as the pLAG3 (data not shown). It is possible that the cytoplasmic domain is not critical for the association between LAG3 and PD1 in CD8^+^ T cells but is required for other aspects of T cell proliferation and function. Detailed mutational analysis in both LAG3 and PD1 is required to delineate the precise location of their interaction.

In summary, the study presented here indicates that LAG3 and PD1 collaborate to mediate T cell function and anti-tumor immunity. LAG3 may enable the rapid translocation of PD1 from early/recycling endosomal compartments to the cell surface (near immunological synapse) via MTOC following T-cell activation and engagement with dendritic cells (or tumor cells). We propose that the binding of LAG3 to MHC I/II molecules and its interaction with adaptor molecules may help stabilize the PD1-PD-L1 ligation and recruitment of SHP2 and SHP1 to regulate the rapid protein phosphorylation occurring at the TCR and IS. Furthermore, in addition to functioning in modulating TCR signaling, LAG3 also plays a role in human monocyte derived DCs [43] and in Treg function [44, 45]. Whether LAG3 and PD1 collaborate in mediating these functions and how LAG3 and PD1 interact with each other on other cell populations warrants further studies.

## MATERIALS AND METHODS

### Mice

C57BL/6 (BL6) mice were purchased from The Jackson Laboratory (Bar Harbor, ME) and bred in our facility (Roswell Park Cancer Institute, RPCI) under an approved Animal Committee protocol. OT-I (H-2K^b^ restricted, anti-OVA TCR transgenic) mice [46] on Rag2^−/−^ background were also bred at RPCI. *Lag3^−/−^* mice [47] were a kind gift from Dario Vignali (St. Jude Children's Research Hospital). *Pdcd1^−/−^* mice [48] were provided by Tasuku Honjo (Osaka University). *Lag3^−/−^*Pdcd1*^−/−^* double knockout mice were generated at the RPCI animal facility. To study the antigen-specific tumor response, all the knockout mice were bred into the OT-1 background. The percentages of the OT-1-transgene were screened by peptide-activation of eye-bled blood cells and stained for the level of the TCRVα2 gene. The tumor rejection experiments described below were performed in C57BL/6 (BL6) mice and/or OT-1 background mice with approximately 40–50% of the CD8^+^ T cells expressing the OVA-TCR transgene. All mice were maintained under specific pathogen-free conditions in the RPCI animal facility according to institutional guidelines. Animal protocols were approved by the RPCI institutional animal care and use committee.

### Cells and culture

OT-1 CD8^+^ T cells were isolated from lymph nodes and/or spleens and activated with plate-bound anti-CD3 antibody (5 μg/ml, BD bioscience) and recombinant B7–1 protein (0.2-0.5 μg/ml, R&D Systems), hereafter called anti-CD3/B7.1, or with SIINFEKL (peptide 326-339, 200 nM) as indicated in the figure legends. For *in vitro* cytokine production study CD8^+^ T cells from C57BL/6, *Lag3^−/−^*, *Pdcd1^−/−^*, and *Lag3^−/−^*Pdcd1*^−/−^* mice were isolated by positive selection using CD8^+^ Flow-comp-Dynal beads (Invitrogen) separation kit and were activated with plate-bound anti-CD3/B7.1 for indicated time. Splenic dendritic cells (DCs) were isolated from C57BL/6 mice and enriched using CD11c^+^ magnetic-activated cell sorting (MACS) beads (Miltenyi Biotec). Purified cells contained approximately 85% CD11c^+^ cells. DCs were cultured with GMCSF (1 μg/ml) and activated with LPS (1 μg/ml) in the presence of SIINFEKL (200 nM) for 20 hours before mixing with T cells to form conjugates. All cells were cultured in RPMI complete media supplemented with 10% fetal bovine serum, 2 mM L-glutamine, 1% minimal essential amino acid, 100 μM sodium pyruvate, 50 μM Δ-mercaptoethanol, 15 mM HEPES, 100 μg/ml penicillin and 100 μg/ml streptomycin.

### Ovarian tumor model

The IE9 cell line, which expresses ovalbumin (OVA) and green fluorescence protein (GFP), was derived from ID8, a mouse ovarian tumor cell line derived from spontaneous *in vitro* malignant transformation of C57BL/6 MOSEC [49] and provided to us by Dr. Tahiro Shin (University of Texas Health Sciences Center, San Antonio, TX). IE9mp1 cell line was generated by *in vitro* expansion of the IE9-OVA orthotopic tumor explant from a C57BL/6 mouse that was injected intraperitoneally. OVA-GFP positive cells were selected by flow cytometry sorting. To generate tumors in a syngeneic mouse model, 6- to 8-week-old female mice were injected intraperitoneally with 1 × 10^7^ IE9mp1cells suspended in 350 μl of PBS. Wild-type C57BL/6 and OT-1 mice bearing the IE9mp1 tumors became moribund at approximately 35-40 and 60-70 days, respectively, after tumor cell implantation. For antibody blockade studies, C57BL/6 mice were randomized and I.P. injected (200 μg per mouse) with IgG control, mouse anti-LAG3 (C9B7W, mIgG1), mouse anti-PD1 (4H2, mIgG1) or anti-LAG3/anti-PD1 every other day (from day 10-18). Blockade treatments were 5 times for survival study and 4 times for TILs and TALs study. All blocking antibodies were gift from Bristol-Myers Squibb. Tumor progression was monitored by the development of abdominal ascites, and analysis of long-term survival. Following institutional guidelines, mice were euthanized when they developed ascites and their abdominal circumference reached 12 cm in diameter or when moribund. Survival (moribund) estimates were calculated with the Kaplan–Meier method and statistics performed with the Mantel-Cox log rank test using GraphPad Prism 6.

### EG7 lymphoma model

EG7-OVA cells was derived from the C57BL/6 (H-2b) mouse lymphoma cell line EL4 [50]. Tumor cells were injected subcutaneously on the left flanks. For antibody blockade treatment, mice were randomized and I.P. injected (200 μg per mouse) with IgG control or indicated antibodies every other day from day 6 to day 12. Tumor growth was measured with an electronic caliper, calculated according to formula, xy^2^/2, and reported as volume (cm^3^). Data shown are from 10 animals per group and representative of three independent experiments.

### Isolation of TALs and TILs

At the indicated time after IE9mp1 tumor challenge, mice were euthanized. TALs were isolated from the ascities through centrifugation and TILs from tumor bed by dissociating tumor tissue in the presence of liberase (25 μg/ml, Roche) and DNase (100 unit/ml, Roche) for 30 min at 37°C. Tissue clumps were removed via filtration through 100 μm mesh. Lymphocytes were also isolated from spleens of non-tumor bearing mice via homogenization and strained through a 100 μm mesh. Red blood cells from all the preparations were removed using ACK lysis buffer.

### Cytokine analysis

CD8^+^ T cells were activated on plate-bound anti-CD3/B7.1 for 6-24 hours. Culture medium were collected and frozen at −70°C until used. The level of IL2, IFN-γ, TNF-α, and Granzyme B in cell culture medium was determined using IL2- or IFN-γ- or TNF-α-or Granzyme B-specific ELISA kits (eBioscience) according to the manufacturer's instructions. For intracellular staining of cytokine production from TILs, cells were incubated with SIINFEKL or with PMA (50 ng/ml) and ionomycin (500 ng/ml) as indicated in the presence of brefeldin (10 μg/ml) for 5 h. Intracellular staining with anti-IL2, anti-IFN-γ, and anti-TNF-α antibodies, was performed as previously described [10].

### Flow cytometry and imagestream analysis

Activated cells were collected at various time points and stained with Fc Block and respective antibodies (see below). The samples were run on a FACS LSRII flow cytometer (BD Biosciences) and analyzed by FCS Express (De Novo Software). For ImageStream analysis, cells were stained as described above. Cells were run on the ImageStream multispectral imaging flow cytometer (AMNIS), and images were analyzed using IDEAS image-analysis software. Briefly, gating was done according to the principle for FACS. Histograms were plotted for each antibody, gating for colocalized events was based on visual inspection of histogram bins, and the mean fluorescence intensities compared. A histogram showing gradient RMS of the bright-field images is drawn to isolate in focus cells. Colocalization was examined by looking at the similarity score of two stained molecules. A mask was drawn to isolate a 5 pixel ring around the entire cell and examined pixel by pixel, and the coincidence of both signals determined, resulting in a similarity score. A histogram is plotted showing the similarity scores for the entire population, and a mean score calculated.

### *In vitro* proliferation assay using CFSE dilution

CD8^+^ TILs were selected using Dynabeads FlowComp CD8^+^ isolation kit (Invitrogen). PD1^−^LAG3^−^, PD-1^+^, LAG3^+^, and PD1^+^LAG3^+^, CD8^+^ TILs were sorted to more than 95% purity by FACS and labelled with CFSE (2.5 μM) for 10 mins and cultured in plates pre-coated with anti-CD3/CD28 antibodies for 4 days. Cells were then stained with anti-CD8 antibody and CFSE dilution of the CD8^+^ T was analyzed using Flow cytometry.

### Confocal microscopy

CD8^+^ T cells from OT-1 mice were activated with plate-bound anti-CD3/B7.1 for 48 h. Cells were washed with PBS and allowed to adhere on charged slides for 5 min fixed with 4% formaldehyde for 15 min, quenched with 125 mM glycine for 5 min. To form DCs and T cell conjugates, activated DCs were stained with Cell Tracker Blue (CTB, Invitrogen) for 45 min, quenched for 30 min, washed with PBS and then mixed with activated CD8^+^ T cells (ratio 1:2) for 20 min before adhering on slide as described above. For staining, cells were permeabilized with 0.2% Triton X-100 for 5 min, washed, blocked with buffer containing 1.5% BSA, 5% donkey serum and 0.05% Tween-20 and stained with indicated primary and secondary antibodies. In some cases, DAPI was included for nuclear counterstain. Images of the stained cells were taken using a Leica confocal microscope. To confirm colocalization, 1 μm Z-sections were reconstructed using ImageJ program [51].

### Immunoprecipitation and immunoblotting

Activated CD8^+^ T cells were rested in IL2 for 2-3 days and washed. Cells (2 × 10^7^) were restimulated with anti-CD3-B7 or IE9mp1 (with or without IFN-γ treatment) and lysed on ice for 30 min, followed by centrifugation. The lysis buffer contained 20 mM Tris pH 7.4, 100 mM NaCl, 1 mM orthovanadate, 10 mM sodium fluoride, 1 mM PMSF, 0.1% Nonidet P-40 (Sigma-Aldrich), 0.1% Triton X-100, and protease inhibitor cocktail (Roche or Sigma). Total extracts were immunoprecipitated with Dynal beads (Invitrogen) conjugated with appropriate antibodies or IgG control antibodies. After washing for 5 times immunoprecipitates were eluted with 1X SDS non-reducing loading buffer, treated with 1/10 volume of 2-ME, and heated at 75°C before loading on a 10% polyacrylamide gel and transferred to a PVDF membrane. In some experiments for detecting SHP2-complex, activated T cells were expanded with IL2 for 3 days and treated with pervanadate (0.024% H_2_O_2_, 160 μM sodium vanadate) in PBS for 5 min at 37°C [36] before lysis. For regular Western blot analysis, the membranes were blocked for 1 h at 42°C in Tris-based saline buffer containing 10 mM Tris, pH 7.5, 150 mM NaCl, and 0.1% Tween (TBST), 5% bovine serum albumin (BSA), probed with specific primary antibodies (in TBST containing 2.5% BSA), washed with TBST and incubated with the appropriate secondary antibodies. Signals were detected with enhanced chemiluminescence (Amersham/GE, or Pierce). For quantitation, scanned images were quantified with ImageJ [51].

### Antibodies

For flow cytometry and ImageStream analysis: phycoerythrin (PE)-conjugated LAG3 (eBioscience), PD1-botin (RMP1–14, Biolegend), PD1-BV421 (Biolegend), (perCP-conjugated CD8a (eBioscience), and FITC-conjugated Thy1.1. For immunoprecipitation: rat-anti-mLAG3 (BD, Pharmingen monoclonal), rat-anti-mPD-1 (a gift from T. Honjo, or eBioscience, Rat IgG2), control rat-IgG1 and IgG2 (Biolegend), control rabbit IgG (Santa-Cruz or Biolegend), rabbit anti-SHP2 (Santa-Cruz or AbCam). For Western blot analysis: goat anti-LAG-3 mAb (C9B7W, R&D Systems), goat anti-PD1 mAb (R&D Systems), anti-SHP1 (AbCam), anti-SHP2 (Santa-Cruz). All the secondary antibodies were from KPL. For confocal microscopy: rat-anti-mPD-1 (RMP1–14, from Tasuku Honjo, Osaka University), goat-anti-LAG3 (R&D Systems), rabbit-anti-EEA1 (Cell Signaling), rabbit-anti-Rab11b (Cell Signaling), rabbit-anti-LAMP1 (Cell Signaling), mouse-anti-TGN38 (BD Biosciences), rabbit-anti-tubulin-r (Biolegend), pLck (Cell Signaling). The following secondary antibodies, absorbed against cross-reactive species, were used: goat-Cy3 and mouse-DyLight-649 were from Jackson ImmunoResearch and rat-Alexafluo-488 and rabbit-Alexafluo-647 antibodies were from Molecular Probe.

### LAG3 and PD1 constructs and retroviral transduction

The LAG3 constructs carried in a murine stem cell virus-based retroviral vector, MSCV-IRES-GFP (pMIG-II), pMIG-LAG3-Wt (pLAG3), or pMIG-LAG3ΔCY (pDCY) and the GP+E86 cells expressing the viral milieu were kindly provided by Dario Vignali (St. Jude Children's Research Hospital). The PD1 constructs (pFB-PD1 and pFB-PDF1F2) carried in pFB vector (Agilent) were provided by Taku Okazaki (The University of Tokushima) and were recloned into the pMIG-II vector, transfected into Platinum-E virus producing cells (Cell Biolabs). Retroviral transduction into T cells was performed as described [22] with minor modification. Briefly, medium from the virus-producing cells were collected and concentrated 10 fold using 100-KDa cut-off spin-columns (Millipore). Concentrated retroviral supernatant supplemented with IL2 (10–20 U/ml, PeproTech) and polybrene (6 μg/ml, Sigma) was added to CD8 T cells that have been activated for 24 h and spin-transduced at 2000 rpm for 60 min. The transduction was repeated once 24 h later. At 72 h after initial stimulation, cells were washed, sorted and expanded in IL2-containing medium for 3 days before harvesting. For some experiments, the retroviral supernatant was first spun onto retronectin-coated plates and the activated T cells along with IL2 were then added and spun for 5 min. The transduction was repeated 24 h later and the cells were sorted, expanded and assayed 2 days later.

## SUPPLEMENTARY FIGURES


