# Supplementary material for: LAG3 and PD1 co-inhibitory molecules collaborate to limit CD8+ T cell signaling and dampen antitumor immunity in a murine ovarian cancer model
Source: Oncotarget. 2015 Jul 23;6(29):27359–77. doi: 10.18632/oncotarget.4751 (PMC4694995; doi:10.18632/oncotarget.4751)
Supplement: Supplementary file 1 [file oncotarget-06-27359-s001.pdf]

SUPPLEMENTARY FIGURES

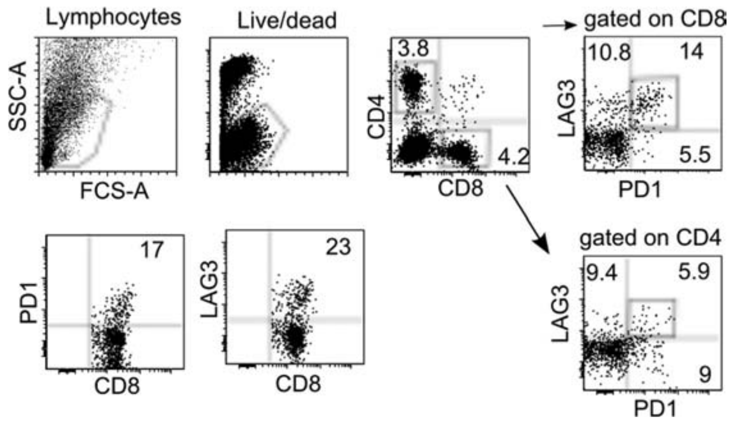

Supplementary Figure S1: Flow cytometry gating for analysis of LAG3 and PD1 expression in TILs and TALs. TILs or TALs were isolated as described in Material and Methods. Stained cells were first gated on lymphocytes based on the FSC-A and SSC-A, then live cells, CD4 and CD8, and then PD1 and LAG3. Numbers indicate percentage of positive cells.

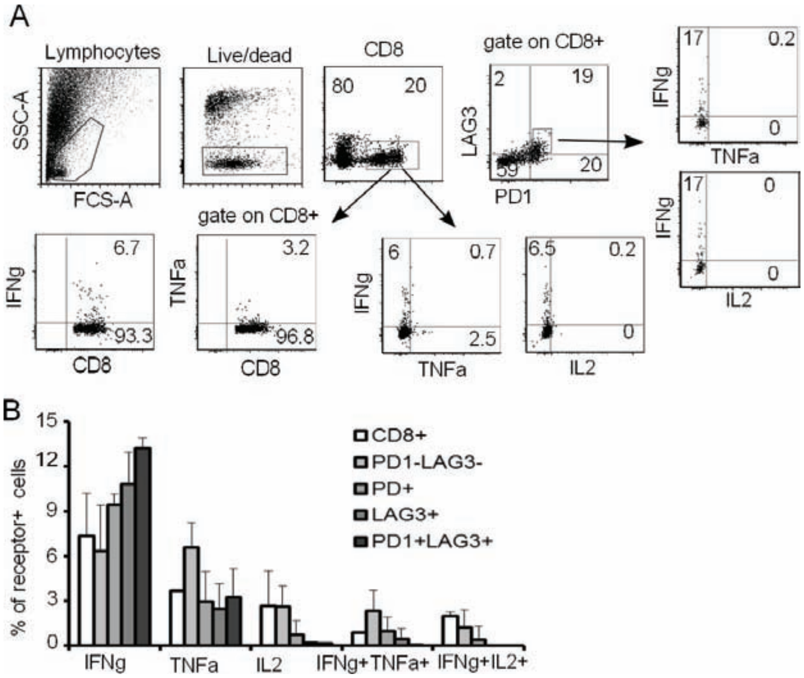

Supplementary Figure S2: Flow cytometry gating for analysis of cytokine production in TILs and TALs. A. Stained cells were first gated on lypmphocytes, live cells and then CD8. CD8$^+$ TILs cells with single and double positive for PD1 and LAG3 staining were then gated for IFN-γ, TNF-α and IL2 or both of IFN-γ and TNF-α or IL2. **B.** Frequencies of single or poly-cytokine producing cells from total CD8$^+$, PD1$^+$, LAG3$^+$, or PD1$^+$ LAG3$^+$ population. TILs were incubated with SIINFEKL for 5 h and stained as described in Materials and Methods. Data shown are average of three mice and are representative of three independent experiments.

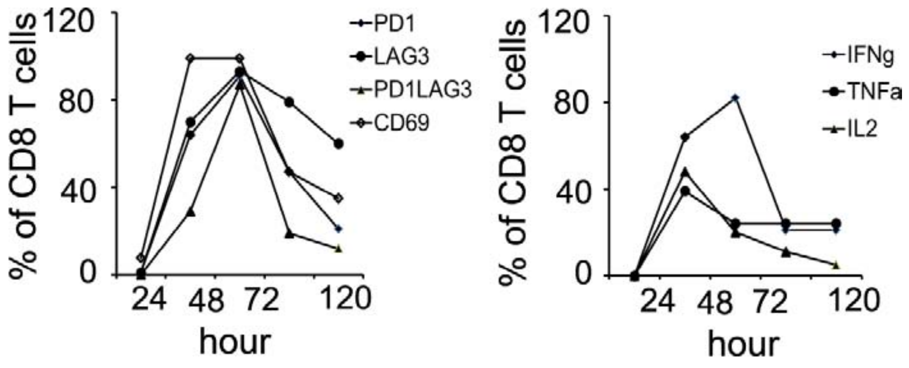

Supplementary Figure S3: *In vitro* activated and rested T cells exhibit exhausted phenotype. CD8 T cells or splenocytes were activated with plate-bound anti-CD3/B7.1 for 48 h and then rested in IL2-containing media (20 U/ml) for 4 days. Cells were collected at the indicated time and stained for the expression of PD1, LAG3, CD69, and the cytokines IFN-γ, TNF-α or IL2. Data shown are representative of three independent experiments.
